# Investigating the link between seed morphology and germination success: insights from European common wild oat (*Avena fatua*) populations

**DOI:** 10.1111/plb.70113

**Published:** 2025-09-28

**Authors:** M. Oveisi, D. Sikuljak, A. A. Anđelković, D. Bozic, P. Poczai, R. Piri, S. Vrbnicanin

**Affiliations:** ^1^ Department of Agronomy and Plant Breeding, College of Agriculture and Natural Resources University of Tehran Karaj Iran; ^2^ Institute for Plant Protection and Environment Belgrade Serbia; ^3^ Institute for Phytomedicine, Faculty of Agriculture University of Belgrade Belgrade Serbia; ^4^ Botany and Mycology Unit, Finnish Museum of Natural History University of Helsinki Helsinki Finland; ^5^ Organismal and Evolutionary Biology (OEB) Research Programme, Faculty of Biological and Environmental Sciences University of Helsinki Helsinki Finland

**Keywords:** *ANN*, *Avena fatua*, geographic location, germination, seed morphology, temperature

## Abstract

- Germination cardinal temperatures, germination rate for 50% of seeds (*GR*_*50*_), and seed traits are interrelated and allow prediction of germination behaviour based on seed characteristics.
- We examined the relationships between seed traits and germination cardinal temperatures in 122 *Avena fatua* populations from 16 European countries, analysing data from 22,000 seeds using image analysis. By germination testing across a temperature range of 5–35*°*C, the germination rate for 50% of seeds (*GR*_*50*_), base temperature (*T*_*b*_), optimal temperature (*T*_*o*_), and ceiling temperature (*T*_*c*_) were estimated using a Dent‐like segmented model.
- A primary response screening analysis revealed that seed colour was the main determinant of *GR*_*50*_. For *T*_*b*_, seed colour and surface hairiness were influential factors, while *T*_*o*_ and *T*_*c*_ were affected by seed colour and the awn attachment point on the lemma. Predictions from artificial neural networks indicated that smaller seeds with shorter awns, wider awn angles relative to the seed axis, higher attachment points on the lemma, and lower surface hairiness are likely to have higher germination rates. Darker‐coloured seeds had higher *T*_*b*_ values than lighter‐coloured seeds. Seeds with awns attached higher on the lemma predominantly had higher *T*_*b*_ values. Black seeds, the most common colour, had a lower *T*_*c*_ than other colours. Considering geographic locations linked to germination cardinal temperatures, seeds from higher latitudes had lower *T*_*b*_ values, and seeds from lower longitudes were predicted to have lower *T*_*c*_.
- This study demonstrated that specific seed morphological traits, such as seed mass, awn length and angle, hairiness, and awn attachment, consistently influence germination performance under varying environmental conditions. These associations suggest adaptive differentiation shaped by both climate pressures and geographic gradients.

## Graphical abstract

Seed traits predict germination success in wild oat: morphology and geography shape thermal thresholds across Europe.

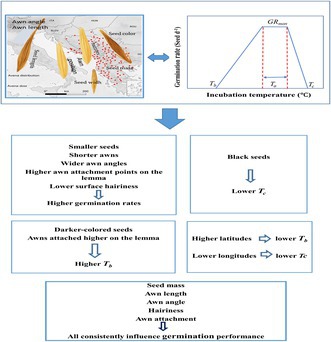

## INTRODUCTION

The Poaceae (or Gramineae) family is one of the largest and most diverse plant families with remarkable adaptability, which contributes to its extensive distribution across the world, including Europe. This family includes grasses, which are highly versatile and resilient and thrive in a wide range of environments, from temperate grasslands and savannas to wetlands, forests, and even arid deserts (Schubert *et al*. 2020). The adaptability of Poaceae species is related to evolutionary development of key morphological traits, such as robust root systems, flexible growth forms, and efficient reproductive strategies, e.g., wind pollination and specialized seed dispersal mechanisms (Linder *et al*. 2018). These traits allow Poaceae to dominate ecosystems by outcompeting other plant families and thriving across diverse climates and soil types.

In Europe, grasses have a foundational role in many ecosystems. They form the basis of agricultural systems as major cereal crops and provide essential habitat and food for wildlife (Carlier *et al*. 2005). Within this family, the most frequent and widespread genera globally and in Europe include *Poa* L., *Festuca* Tourn. Ex L., *Lolium* L., *Bromus* L., *Avena* L., *Alopecurus* L., *Agropyron* Gaertn., and *Phragmites* Adans. (Dostatny *et al*. 2021). The *Avena* genus, which includes common wild oat (*Avena fatua* L.), is particularly prevalent in temperate agricultural regions. *Avena* species are significant because of their impact as weeds of cereal crops (Beckie *et al*. 2012; Vrbničanin 2017). *Avena fatua* has several traits that contribute to its success as a widespread and persistent species, particularly in agricultural landscapes. These traits include high adaptability (Sharpe *et al*. 2024), competitive growth (Travlos & Giannopolitis 2010; Vrbničanin 2017), and specialized seed characteristics (Oveisi *et al*. 2024) that enhance its ability to disperse, survive, and establish in diverse environments. One notable feature is seed shedding behaviour; *A. fatua* seeds typically ripen and shed 2–3 weeks before cereal crops mature, ensuring that a significant portion of the seed reserve is returned to the soil before harvest (Vrbničanin *et al*. 2011).

Another key adaptation is *A. fatua* seed dormancy. Seeds that are dormant do not germinate once embedded in the soil; rather, their longevity in soil is regulated by morphological and physiological traits (Ņečajeva *et al*. 2021). Additionally, *A. fatua* is a prolific seed producer. Under favourable conditions, a single plant with 20 shoots can produce up to 1500 seeds (Morrow & Gealy 1982). This high seed output significantly increases its potential for successful establishment and spread, particularly in both arable and non‐arable lands, where it quickly colonizes open spaces (Mahajan *et al*. 2022).

Seeds of *Avena* species are typically elongated, a feature that facilitates their movement and positioning within the soil. Their size provides sufficient energy reserves to support early seedling growth, even in competitive environments. In addition, the colour and texture of *Avena* seeds contribute to camouflage, reducing predation by blending into the soil or surrounding vegetation (Oveisi *et al*. 2024). In some cases, seed colour also influences response to environmental cues, such as light and temperature, which are critical for determining germination timing (Atis *et al*. 2011).

One of the most distinctive morphological traits of *Avena* species is the placement of awns on the lemma (Oveisi *et al*. 2024). Efficient awn positioning can help situate seeds in favourable conditions within the soil, thereby enhancing germination success (Chauhan & Johnson 2010). The seeds of *A. fatua* also have durable seed coats that provide protection from physical damage, desiccation, and predation (Mahajan & Chauhan 2021). This resilience allows the seeds to survive harsh environmental conditions and ensures they remain viable until they encounter suitable conditions for germination.

Morphological seed traits, such as awn presence, tough seed coat, size, shape, and surface characteristics, are essential for the evolutionary success and adaptability of *A. fatua*. These traits enable this species to thrive in a wide range of environments, from agricultural fields to natural grasslands, contributing to its persistence as a significant weed in temperate regions (Cochrane *et al*. 2015). The link between these traits and germination cardinal temperatures and 50% germination (*GR*_*50*_) reflects evolutionary pressures that have shaped *A. fatua* to optimize survival and reproduction in diverse and challenging environments (Wildeman 2004). These adaptations ensure that seeds not only survive adverse conditions but also germinate under the most favourable circumstances, maximizing the likelihood of successful seedling establishment.

Environmental factors act as selective pressures, “sieving” populations to favour individuals with traits best suited for survival and reproduction under specific conditions. To our knowledge, the relationships between seed morphology and germination cardinal temperatures of *A. fatua*, such as base temperature (*T*_*b*_), optimal temperature (*T*_*o*_), and ceiling temperature (*T*_*c*_), have not been experimentally investigated.

In this study, we collected seeds from 122 populations of *A. fatua* from various regions and altitudes across Eastern Europe. Using image‐processing, we derived seed morphological traits (Oveisi *et al*. 2024) and tested germination across a range of temperatures to estimate germination cardinal temperatures. We investigated (i) how individual seed traits, or their interactions, influence germination cardinal temperatures; (ii) which morphological traits are associated with enhanced or reduced germination; (iii) whether specific traits serve as indicators for predicting germination cardinal temperatures; and (iv) correlations between seed morphological traits, germination behaviour, and the geographic origin of the seeds.

## MATERIAL AND METHODS

**Fig. 1:**
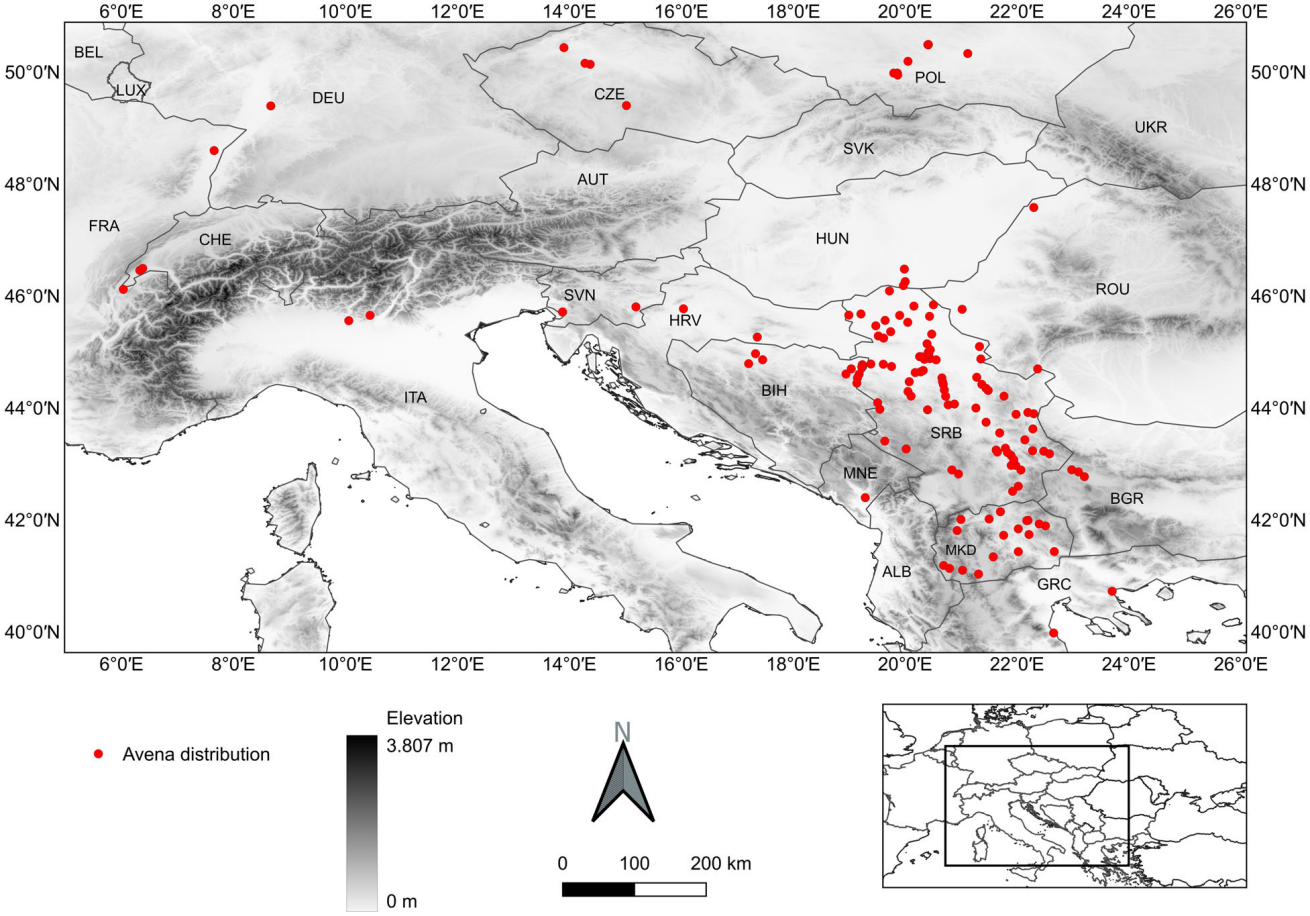
Geographic distribution of collection sites for *Avena fatua* populations analysed in this study.

Seed samples of *A. fatua* were collected in 2019 from 122 sites across Eastern Europe following procedures of Oveisi *et al*. (2024). Collections encompassed 63 sites in Serbia, three in Bulgaria, three in Romania, four in the Czech Republic, and 19 across Poland, Slovenia, and Bosnia and Herzegovina. Additional samples were obtained from North Macedonia, Montenegro, Hungary, Greece, and Croatia and from Italy, France, Switzerland, and Germany (Fig. 1). At the time of seed collection, seeds were fully mature with a moisture level of 9%–11% for all populations sampled. All sampled collection sites were wheat fields. To release physiological dormancy, the seeds were stored in brown paper bags (one large bag per population) at room temperature in the dark for ca. 9 months following the collection date. At each collection site, sampling involved collecting seeds from 10 to 15 *Avena* plants randomly distributed throughout the population, thus achieving the most representative sampling of the population.

Seed images were captured using a Stereo Trinocular microscope equipped with a digital camera following the setup described in Oveisi *et al*. (2024). Morphological analysis included measurements of seed length, width, area, hairiness, awn length, awn angle, and awn attachment point. From each sampled population, 30 seeds were measured for morphological parameters. Seeds were chosen randomly from each population collection bag. We used individual measurements for analysis, not the average. These measurements were performed using ImageJ software with the “Seed Analysis” and “Colour Threshold” plugins. Comprehensive descriptions of the methodologies, covering image preprocessing, seed segmentation, and calibration, are available in Oveisi *et al*. (2024).

### Germination testing

For the germination tests, seeds were placed in Petri dishes in three replicates of 50 seeds per dish for each temperature treatment (5–35°C). Germination was assessed based on radicle protrusion, with seeds classified as germinated upon visible emergence of a radicle of 2 mm in length. Seeds that failed to germinate within the 14‐day observation period were recorded as non‐germinated, provided they were neither empty nor infested by insects. The seeds were germinated in the dark and the bottom of each Petri dish contained a fully saturated circular filter paper. Petri dishes were sealed with parafilm to prevent moisture loss. Seed germination was counted over 336 h (14 days) at 24‐h intervals in a germinator containing separate germination chambers (RUMED, Rubarth Apparate). The cumulative germination fraction (*CGF*) with time was calculated for further analysis:

$${\mathrm{CGF}}_{t}\;=\;\frac{N_{t}}{N_{\text{total}}}$$

where, *CGF*_t_ = cumulative germination fraction by time *t*; *N*_t_ = cumulative number of germinated seeds by time *t*; *N*_total_ = total number of seeds tested.

### Statistical methods

The *CGF* of the populations was described with germination time using a Weibull function. The time to 0.5 of final *CGF* (*T*_*50*_) was then calculated using the *ED()* function in the *drc* package in *R* (v. 2023.03.1). The inverse of *T*_*50*_ (*GR*_*50*_) was the germination rate used for estimating *T*_*b*_, *T*_*o*_, and *T*_*c*_ of germination using a segmented model:

$$\text{If}\;T>T_{c}\;\text{or}<T_{b},0\;\left(\text{zero}\right)$$

$$\text{If}\;\left(T_{c}>T>T_{o}\right),1-\left(T_{c}-T\right)/\left(T_{c}-T_{o}\right)$$

$$\text{If}\;\left(T_{o}>T>T_{b}\right),\left(T-T_{b}\right)/\left(T_{o}-T_{b}\right)$$

The thermal germination range (*TGR*) for each population was calculated as the difference between ceiling temperature (*T*_*c*_) and base temperature (*T*_*b*_). We then investigated the response of germination cardinal temperatures to the measured seed traits, in addition to the longitude, latitude, and altitude, using the response screening method. The influence of the factors was assessed using Log‐worth values, which are defined as the ‐*log*_*10*_ *P*‐value. This transformation adjusts the *P*‐values to provide an appropriate scale for comparing effects of the experimental factors. A *P*‐value > 2 is considered significant at the 0.01 level (log base 10 of 0.01 = 2).

We employed artificial neural networks (*ANN*) to predict *GR*_*50*_ and germination cardinal temperatures (*T*_*b*_, *T*_*o*_, and *T*_*c*_) using a comprehensive set of seed morphological traits. The dataset was randomly divided into three subsets: 45% for training, 30% for validation, and 25% for final model testing. During training, weights and biases were iteratively updated using stochastic back‐propagation (stochastic gradient descent) with a fixed learning rate of 0.1. The input features included seed mass, seed length, fruit width, fruit length, awn length, awn angle, awn contact point on the lemma, percentage hairiness, mean length of three hairs, and seed colour. Seed colour was retained as it demonstrated an association with geographic variation in prior research (Oveisi *et al*. 2024). Before model fitting, multicollinearity among predictors was assessed using Spearman's correlation coefficients. Traits showing correlations >0.7 were excluded to reduce redundancy and overfitting. Each *ANN* model consisted of two hidden layers, with activation nodes chosen from hyperbolic tangent (TanH), linear, and Gaussian functions. Importantly, the architecture was optimized separately for each predicted *GR*_*50*_, *T*_*b*_, *T*_*o*_, and *T*_*c*_, as different traits responded best to different combinations of activation functions. For instance, *GR*_*50*_ was predicted using a configuration of 6 TanH, 2 Linear, and 11 Gaussian nodes in the first layer, and 8 TanH, 2 Linear, and 18 Gaussian nodes in the second layer. Other response variables (*T*_*b*_, *T*_*o*_, and *T*_*c*_) required adjusted compositions of nodes to best capture their non‐linear relationships with the input traits. Each model underwent 10 optimization attempts (tours) starting from randomized initial weights to improve convergence and avoid local minima. The model that minimized prediction error on the validation set was selected as the final model. Model performance was assessed on training, validation, and test datasets using the coefficient of determination (*R* ^2^), root average square error (*RASE*), mean absolute deviation (*MAD*), sum of squared errors (*SSE*), negative log‐likelihood, maximum likelihood (*ML*), and misclassification rate.

Statistical analysis and modelling were conducted in *R*‐studio (v. 2023.03.1) and JMP® pro v. 17.1 (SAS Institute, Cary, NC, 1989–2023). These statistics were used to evaluate both fit and predictive performance, ensuring that the model architecture was robust and generalizable across different subsets of data.

## RESULTS

**Fig. 2:**
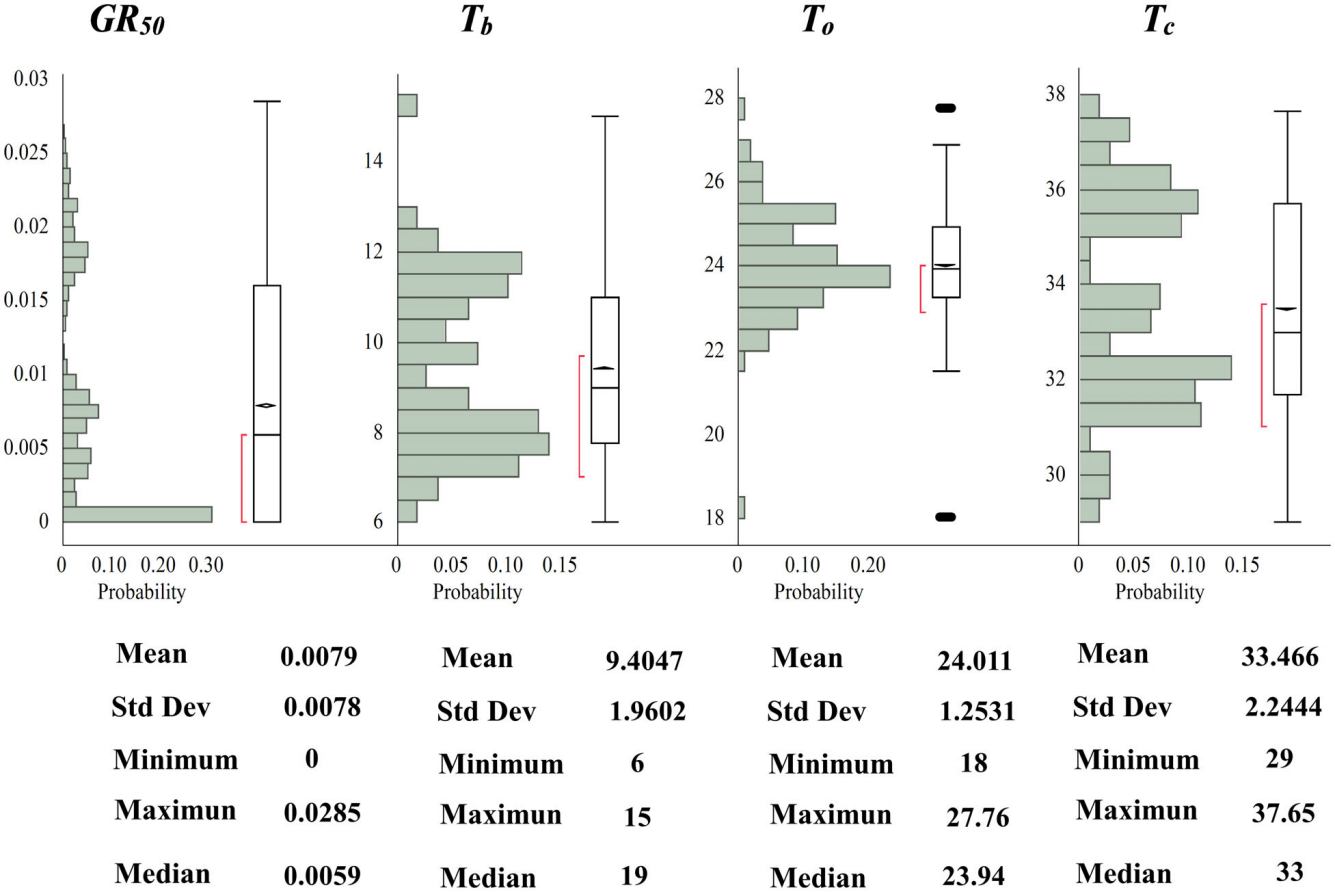
Distribution of *GR*_*50*_, *T*_*b*_, *T*_*o*_, and *T*_*c*_ values estimated using the Dent‐like segmented model. Summary metrics (mean, range, and ±SD) are provided for reference.

**Fig. 3:**
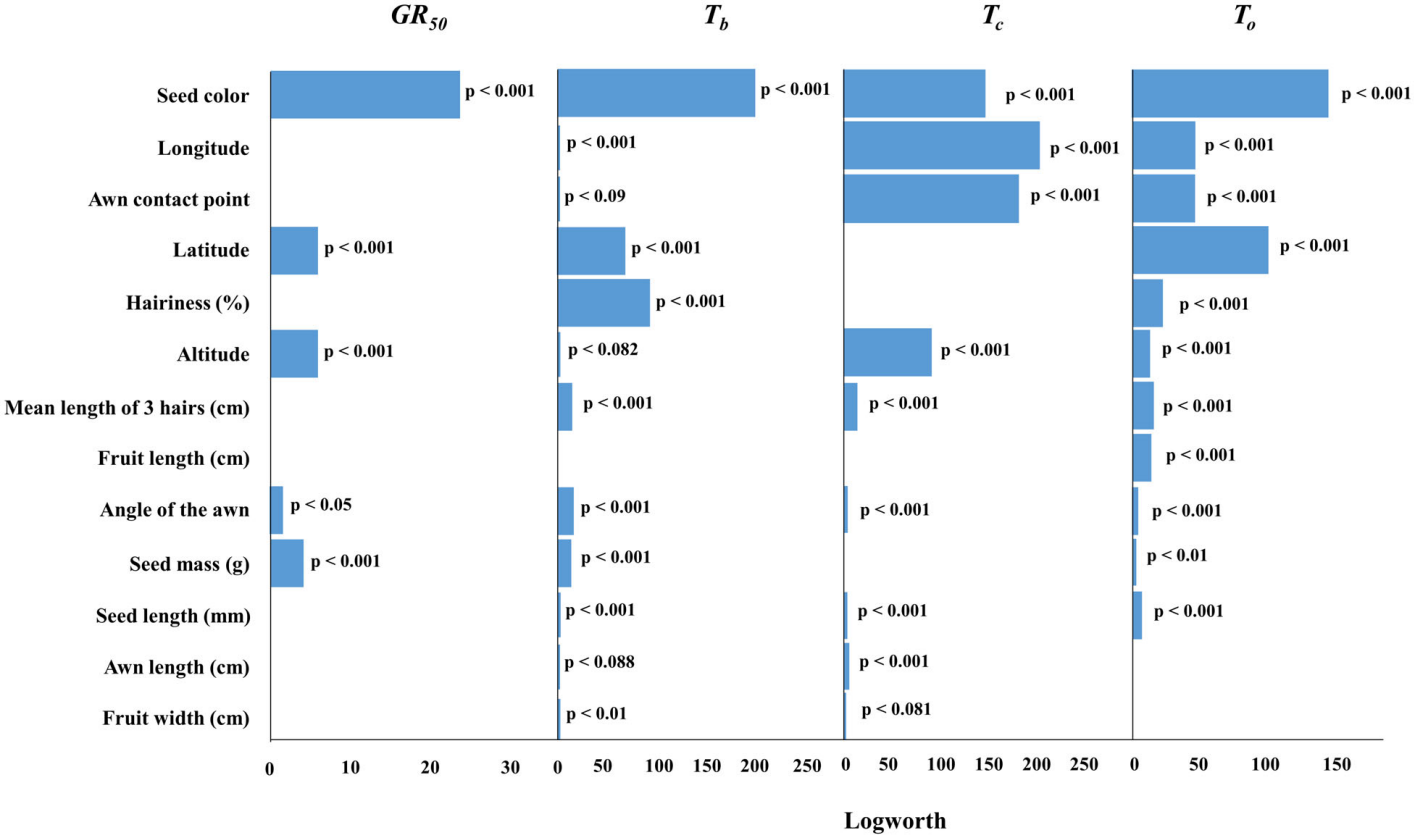
Contributions of seed traits and geographical coordinates for GR50, Tb, To, and Tc values. Higher log‐worth values indicate stronger predictor influence.

Geographic coordinates and altitude significantly contributed to the germination cardinal temperatures of *A. fatua*. Latitude and altitude were the most influential factors in *GR*_*50*_, while latitude also had a strong effect on base temperature (*T*_*b*_) and optimum temperature (*T*_*o*_). In contrast, longitude emerged as the most important geographic predictor of ceiling temperature (*T*_*c*_). Histograms for *T*_*b*_, *T*_*o*_, *T*_*c*_, and *GR*_*50*_ (Fig. 2), which were generated from estimates obtained using a Dent‐like segmented model. The mean *GR*_*50*_ was 0.0078 seed h^−1^ (SD = 0.0078, range 0.006–0.028) and mean *T*_*b*_ was 9.4*°*C (SD = 1.96, range 6–15). Mean *T*_*o*_ was 24*°*C (SD = 1.25, range 18–27.76). Mean *T*_*c*_ was 33.46*°*C (SD = 2.24, range 29–37.65). The *R* ^2^ and *RMSE* values for fitting the Dent‐like segmented model ranged from 0.77 to 0.97, and from 0.001 to 0.02, respectively. Morphological seed traits were also strongly associated with germination cardinal temperatures based on log‐worth values (Fig. 3). *GR*_*50*_ was primarily influenced by seed colour, while *T*_*b*_ was mostly affected by seed colour, followed by hairiness and awn angle. Both *T*_*o*_ and *T*_*c*_ were strongly influenced by awn contact point and seed colour. Additional morphological features—hair length and percentage hairiness—also had significant effects, influencing *T*_*o*_ and *T*_*c*_, respectively. These results demonstrate that both geographic origin and seed morphology play key roles in shaping germination responses of *A. fatua*, with distinct predictors influencing different components of the germination temperature profile. This integrated pattern highlights the interaction between environmental selection pressures linked to location and intrinsic seed traits that contribute to population‐level variation in germination performance.

### Relationship between seed traits and germination rate

**Fig. 4:**
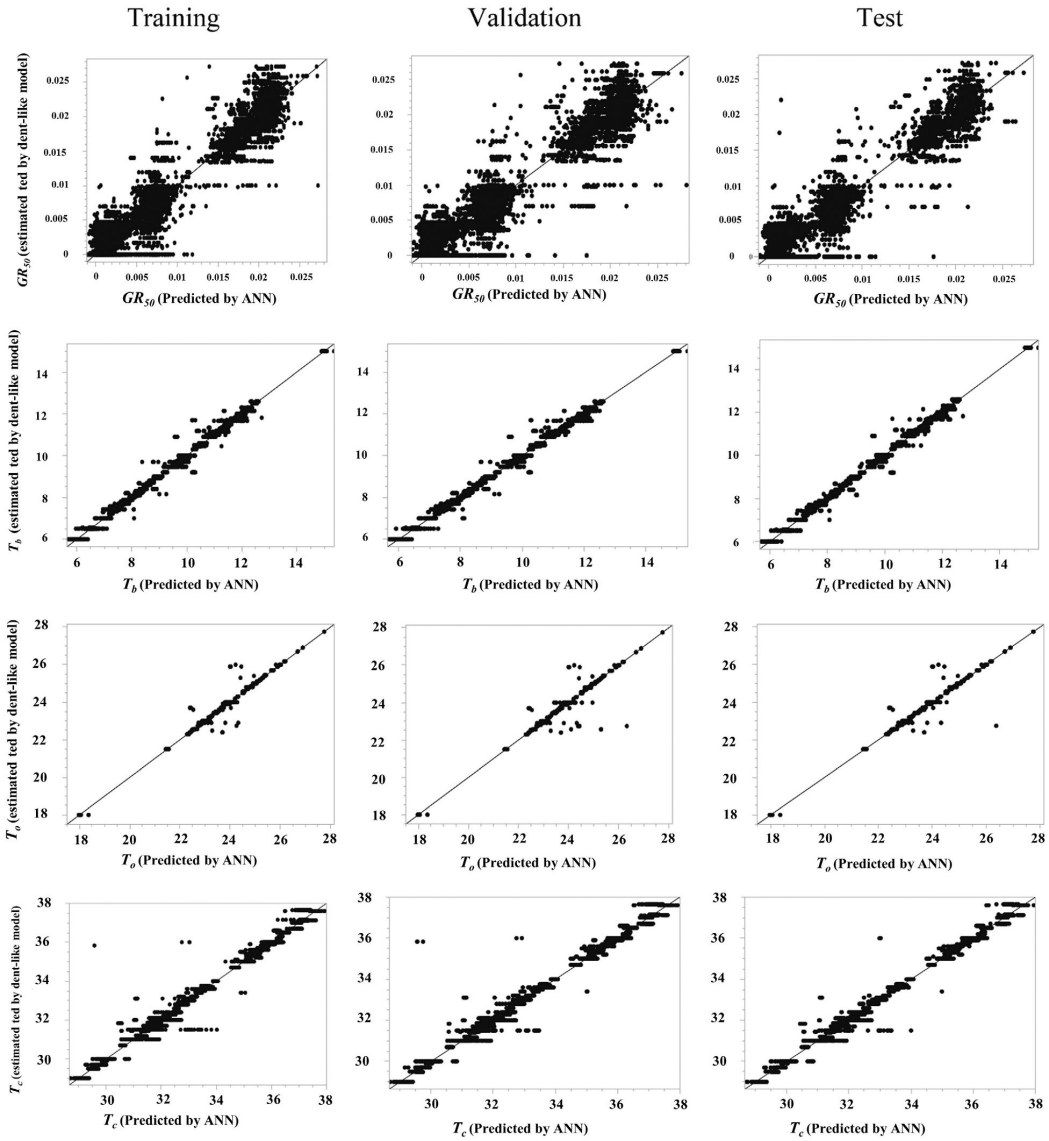
Pairwise comparison of *GR*_*50*_, *T*_*b*_, *T*_*o*_, and *T*_*c*_ values predicted by the ANN model and those derived from the Dent‐like segmented model, considered as actual values.

The *ANN* model predicted *GR*_*50*_ values based on input variables, achieving an *R* ^2^ of 0.96 and an *RMSE* of 0.002 during training. For both validation and testing, the model yielded an *R* ^2^ of 0.94 and an *RMSE* of 0.002. One‐to‐one line plots for training, validation, and test sets revealed strong agreement between observed and predicted values (Fig. 4).

**Fig. 5:**
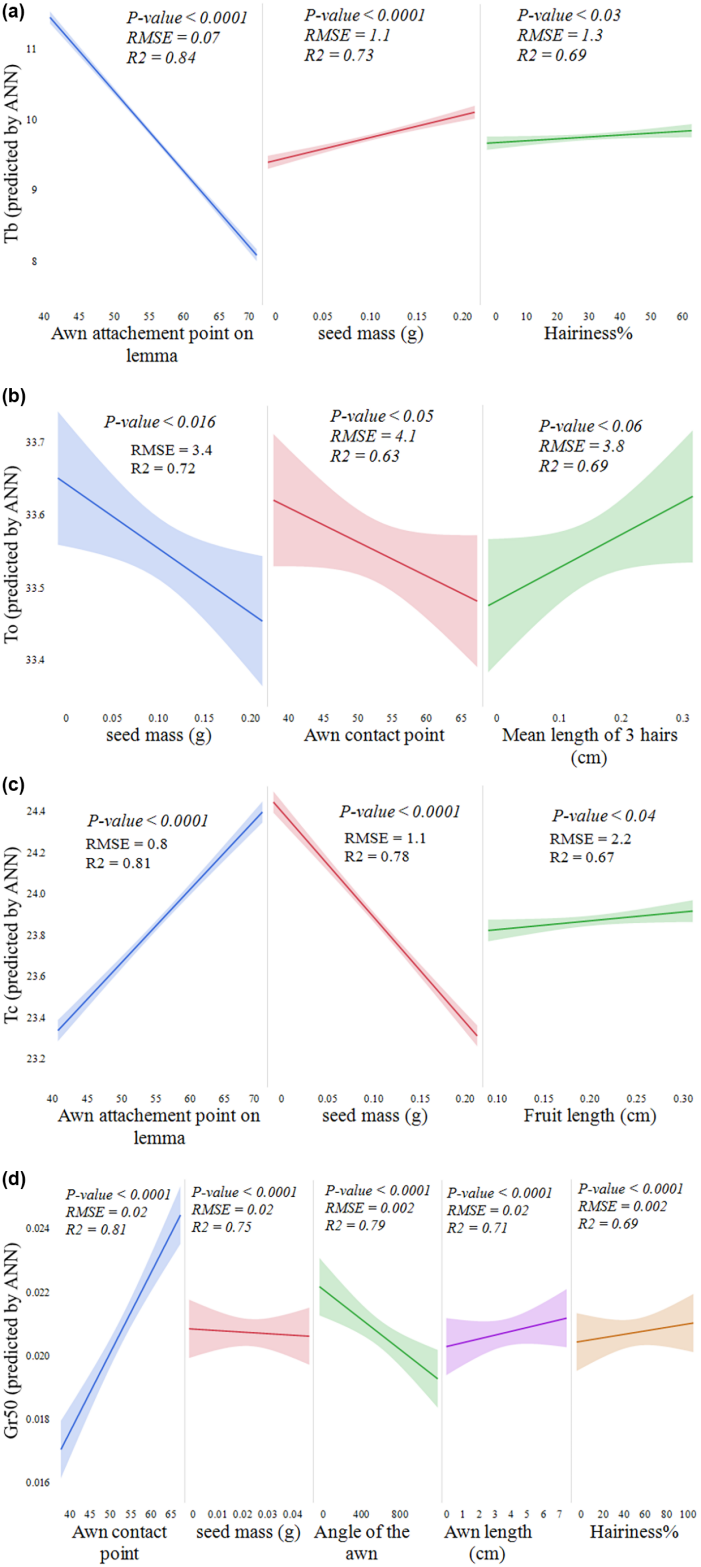
Seed trait influences on thermal thresholds and germination rate predicted by ANN. Relationships between (a) base temperature (*T*_*b*_), (b) optimum temperature (*T*_*o*_), (c) ceiling temperature (*T*_*c*_), and (d) germination rate at 50% completion (*GR*_*50*_) and the seed morphological traits that significantly contributed to their prediction by the artificial neural network (ANN) model. In each panel, regression lines are shown with 95% confidence intervals (shaded areas), along with *P*‐values, coefficient of determination (RC), and root mean square error (RMSE) for each relationship.

Additionally, there were significant linear relationships (*P* < 0.01) between *GR*_*50*_ and seed traits, such as seed mass, awn angle, awn contact point on the lemma, and seed surface hairiness (Fig. 5). *GR*_*50*_ decreased with increasing seed mass, indicating a lower germination rate for larger seeds. *GR*_*50*_ also increased with a larger awn angle, decreased with awn length, decreased with a higher awn contact point on the lemma, and decreased with increased seed surface hairiness. Therefore, smaller seeds with shorter awns, larger awn angles relative to the seed axis, attachment at the upper part of the lemma, and lower surface hairiness are expected to have higher germination rates.

### Linking seed traits to base, optimum, and ceiling temperatures

The *ANN* accurately predicted *T*_*b*_, *T*_*o*_, and *T*_*c*_ across training, validation, and testing phases. For *T*_*b*_, *R* ^2^ > 0.98 with *RMSE* < 0.12; for *T*_*o*_, *R* ^2^ > 0.96 with *RMSE* < 0.9; and for *T*_*c*_, *R* ^2^ > 0.99 with *RMSE* < 0.01. These metrics confirm the strong performance and reliability of the *ANN* predictions. The model predicted that shifting the awn attachment point through the lemma tip correlates with a decrease in *T*_*b*_, while increases in seed mass and hairiness percentage correlate with an increase in *T*_*b*_. For *T*_*o*_, the awn placement through the lemma tip similarly correlated with decreasing *T*_*b*_ and was also associated with a decrease in *T*_*o*_ as seed mass increased. Longer hairs were predicted to correlate with an increase in *T*_*o*_. For *T*_*c*_, placement of the awn through the lemma tip was predicted to increase *T*_*c*_, while higher seed mass decreased *T*_*c*_. Fruit length was also predicted to increase *T*_*c*_, although this was less influential (Fig. 5).

### Geographic patterns in seed colour and germination temperature

**Fig. 6:**
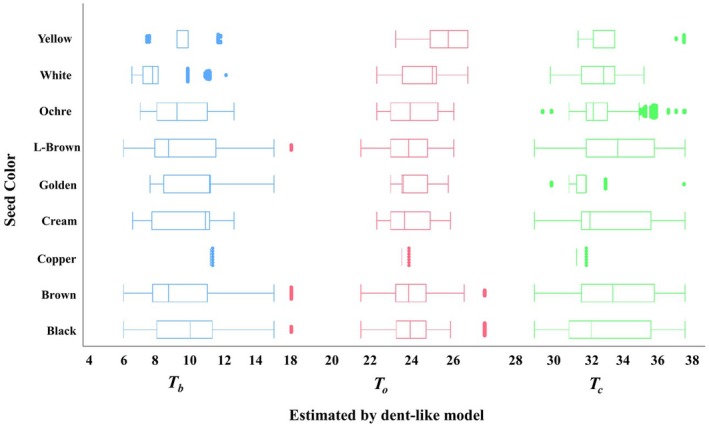
Distribution of base, optimum, and ceiling germination temperatures among seeds of different colours, as estimated by the Dent‐like segmented model. Boxplots show the median, interquartile range, and variability within each seed colour category, with outliers plotted as individual points.

Seed colour was associated with distinct differences in *T*_*b*_, *T*_*o*_, and *T*_*c*_ (Fig. 6). *T*_*b*_ and *T*_*c*_ exhibited greater variation than *T*_*o*_, with black, cream, and golden seeds having the highest mean *T*_*b*_ values, and light brown and brown seeds the lowest. Yellow and white seeds had higher mean *T*_*o*_ compared with other colours, while variation in *T*_*o*_ was otherwise limited. For *T*_*c*_, black seeds had the lowest mean values, whereas brown and light brown seeds had the highest.

**Fig. 7:**
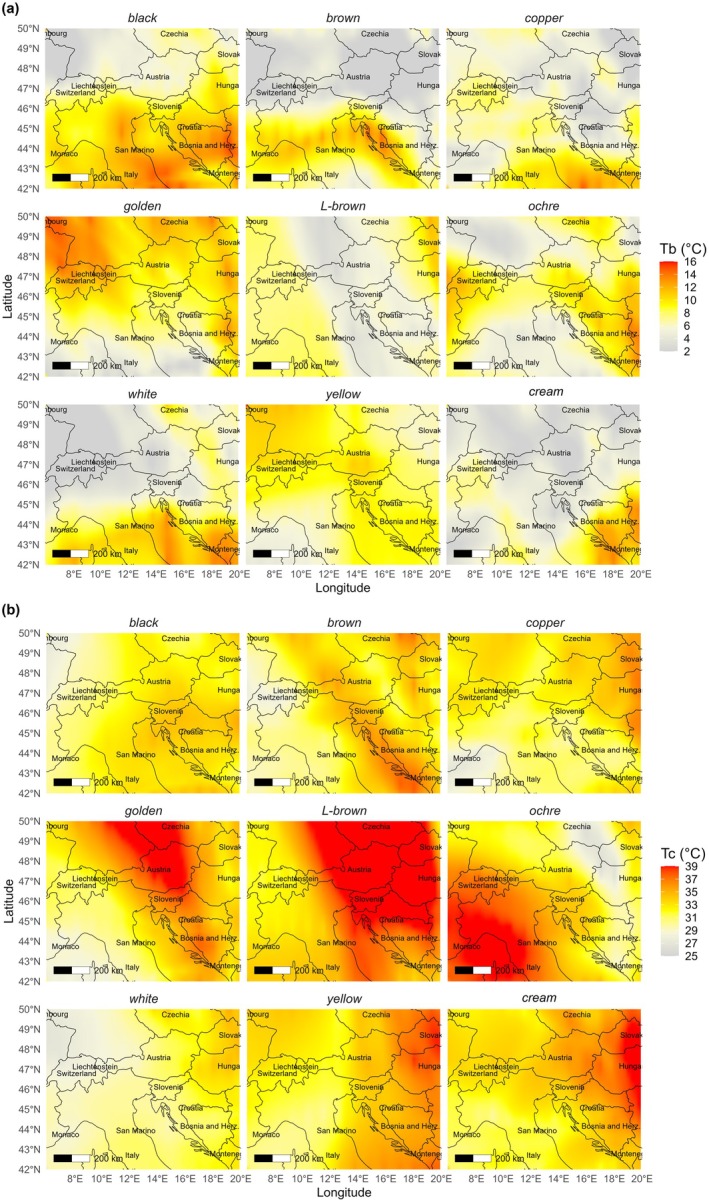
Spatial gradients of thermal thresholds across the seed collection area predicted by the ANN model. (a) Geographic distribution of base temperature (*T*_*b*_) for seeds of different colours, showing variation from cooler (grey/white) to warmer (yellow/red) thresholds across the sampling region. (b) Geographic distribution of ceiling temperature (*T*_*c*_) for seeds of different colours, illustrating regional differences in upper thermal limits. Maps are based on ANN‐predicted values and plotted for each seed colour category, with colour scales indicating the respective temperature ranges.

To explore spatial patterns, seed colour, altitude, longitude, and latitude were included as predictors in ANN models for *T*_*b*_, *T*_*o*_, *T*_*c*_, and *GR*_*50*_. High predictive accuracy was achieved only for *T*_*b*_ and *T*_*c*_. Mapping the ANN outputs (Fig. 7) showed that both parameters varied geographically in relation to seed colour. In general, seeds from higher latitudes had lower predicted *T*_*b*_, except for cream and yellow seeds, which showed the opposite trend. For *T*_*c*_, seeds from lower longitudes were predicted to have lower values, and those from higher longitudes higher values, again with cream‐coloured seeds displaying an inverse pattern.

**Fig. 8:**
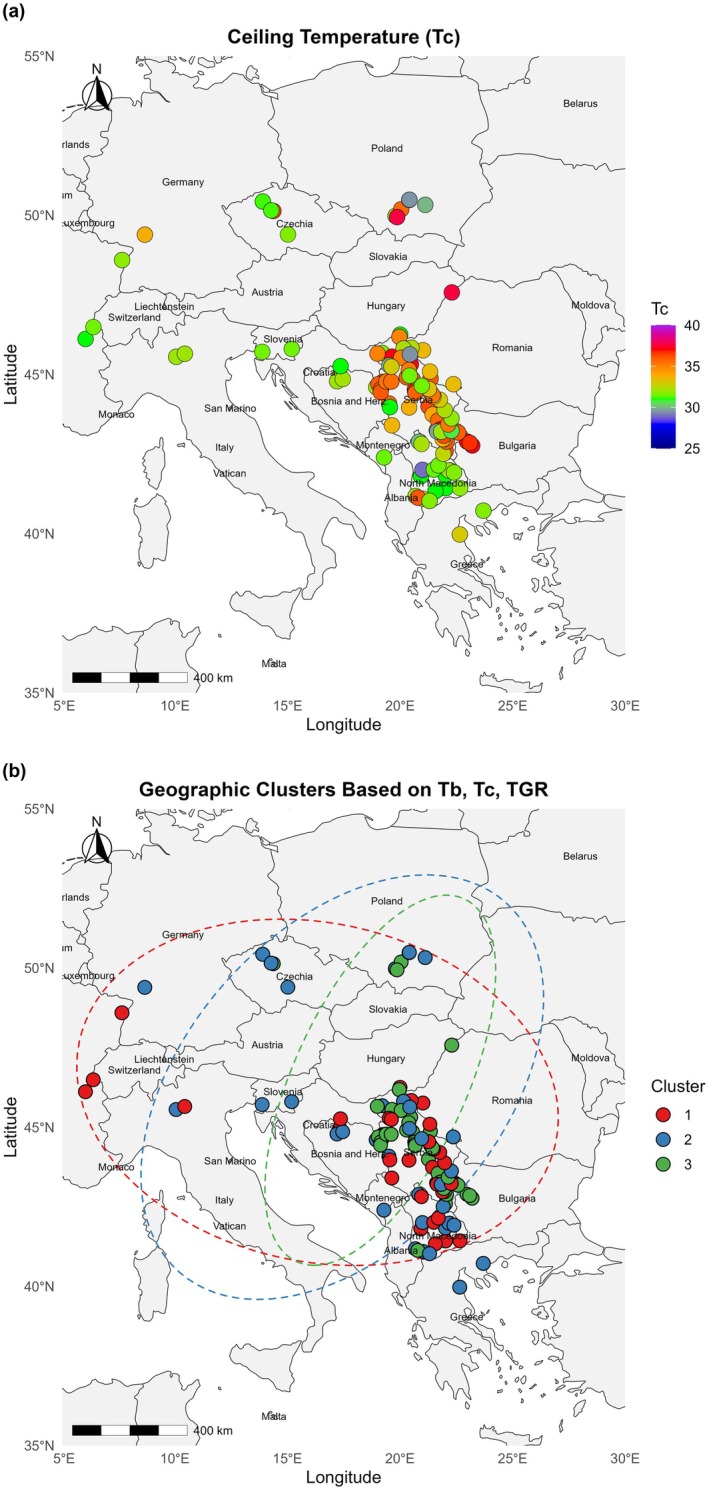
Geographic patterns of thermal germination range (TGR, °C) and population clustering in *Avena fatua* across southeastern and central Europe. (a) Spatial variation in TGR, with colours indicating magnitude. (b) Hierarchical clustering of populations based on observed thermal parameters; colours denote cluster identity and dashed ellipses outline approximate cluster extents.

Thermal germination range (TGR) varied markedly among populations, spanning 10.2–31.7°C with a mean of 21.3 ± 4.9°C. Broader TGR values were concentrated in eastern Serbia, central Romania, and northern Bulgaria, whereas narrower ranges predominated in western Austria, Slovakia, and northern Greece. A moderate positive correlation between TGR and longitude (Pearson's *r* = 0.58, *P* < 0.01) indicated an eastward increase in TGR, a pattern consistent with underlying climate gradients, although direct environmental predictors were not assessed in this analysis (Fig. 8a).

Cluster analysis of four thermal germination traits (*T*_*b*_, *T*_*c*_, *GR*_*50*_, and *TGR*) identified four statistically distinct and internally coherent groups (silhouette index = 0.73), each exhibiting spatial structure and regional differentiation (Fig. 8b). Populations from the central Balkans (Cluster 1) were characterized by high *T*_*c*_, broad TGR, and elevated *GR*_*50*_, consistent with a comparatively broad thermal niche. Southern Balkan populations (Cluster 2) displayed higher *T*_*b*_ and narrower TGR, suggesting more limited thermal tolerance. A third group, spanning western and central Europe (Cluster 3), showed intermediate values for all traits, reflecting a conservative germination strategy. The final cluster, from the northeastern Carpathian Basin and neighbouring northern Romania and Ukraine (Cluster 4), had lower *T*_*c*_, moderate *T*_*b*_, and restricted TGR. The spatial coherence of these clusters underscores a geographic basis for differentiation in germination thermal responses, potentially shaped by regional climate conditions.

## DISCUSSION

### Differences in germination cardinal temperatures between populations

There was a broad range of germination cardinal temperatures (*T*_*b*_, *T*_*o*_, and *T*_*c*_) and *GR*_*50*_ across different *A. fatua* populations, with values for *T*_*b*_, *T*_*o*_, and *T*_*c*_ from 8 to 9*°C*. These variations are likely related to genetic differences (Li *et al*. 2007), parental effects (Aghabeigi *et al*. 2016), and morphological and physiological seed traits (Ņečajeva *et al*. 2021). The populations used in this study were collected from diverse locations spanning longitudes of 6°–24°, latitudes of 37°–49°, and altitudes of 100–800 m. These regions vary widely in bioclimate conditions (Ðurᵭević *et al*. 2023), soil properties (Orgiazzi *et al*. 2018), land cover, biodiversity (Schirpke & Tasser 2021), and microbiomes (Labouyrie *et al*. 2023), which can all contribute to the observed variability in germination cardinal temperatures.

### Germination rate ( *GR*_*50*_ ) and seed traits

The *GR*_*50*_ values tended to decrease with increasing seed mass, indicating that larger seeds had slower germination rates. Larger seeds often contain more stored resources, which can lead to slower metabolic activation but increased resilience in challenging environments (Souza & Fagundes 2014; Khan *et al*. 2024). Additionally, larger seeds typically have a thicker or harder seed coat that provides extra protection but can also slow water absorption (Upretee *et al*. 2024).

The model also predicted that seeds with shorter awns, awns attached higher on the lemma, and lower surface hairiness tend to have higher germination rates. Initially, we hypothesized that smaller awns might correlate with smaller seed mass, but neither Spearman correlation nor variance inflation factor *(VIF)* tests showed strong correlations (values < 0.2). However, smaller lemmas might correspond with thinner seed coats, potentially facilitating faster water absorption and contributing to higher *GR*_*50*_ values. Seeds with less hairiness can also absorb water more evenly, which supports more rapid germination, as hairs can act as a barrier that delays water uptake (Glison *et al*. 2017). Further, seeds with awns attached lower on the lemma are generally larger and have more hairs (Oveisi *et al*. 2024), both of which are associated with slower water absorption and thus a lower germination rate (Glison *et al*. 2017; Vidak *et al*. 2022). In general, several seed coat traits, including size, thickness, and differences in anatomical structure (e.g., presence of pores or cuticles) alter water permeability and affect *GR*_*50*_ (Upretee *et al*. 2024).

### Influence of awn position, seed mass, and hairiness on base temperature

Shifting the awn towards the lemma tip was associated with a decrease in *T*_*b*_. Additionally, higher seed mass and hairiness appeared to contribute to this decrease, likely representing an adaptive strategy to lower *T*_*b*_. Higher seed mass and hairiness enhance seed tolerance to environmental stresses, while a more distal awn position facilitates water absorption, potentially related to a thinner seed coat (Ribeiro *et al*. 2015; Upretee *et al*. 2024). This adaptation could help seeds retain moisture, regulate temperature under diverse environmental conditions, and promote germination at lower temperatures.

### Adaptation traits for heat tolerance

Certain seed traits, such as longer hairs, are associated with an increase in *T*_*o*_. In contrast, *T*_*o*_ tends to decrease with increasing seed mass, and *T*_*c*_ also tends to decrease as seed mass increases. Larger seeds generally retain heat for longer because of their lower surface‐to‐volume ratio (Souza & Fagundes 2014), while longer hairs help prevent overheating (Kaur & Kariyat 2020). Smaller seeds, which absorb water quickly, may release excess heat more effectively, facilitating efficient heat exchange and better temperature regulation under high temperatures (Notarnicola *et al*. 2023). Additionally, longer hairs help retain moisture for extended periods, which mitigates overheating and maintains germinability at elevated temperatures (Robinson *et al*. 2008). Taken together, these traits may act as heat regulators for the seed, with elongated seeds supporting efficient heat dissipation, which allows them to remain viable across a broader temperature range (Moosavi *et al*. 2022).

### Why was ceiling temperature a better predicted than other parameters?

The prediction of *T*_*c*_ had higher accuracy than that of other parameters. Generally, *T*_*c*_ is a conservative parameter that ensures prolonged germination at higher environmental temperatures (Balouchi *et al*. 2023). In contrast, *T*_*b*_ controls initiation of germination as soon as environmental conditions are suitable. While *T*_*b*_ is time‐sensitive, early‐season temperatures are highly variable from year to year. Consequently, seeds have evolved a broad range of *T*_*b*_ values across populations, ensuring germination occurs at the optimal time (Amin *et al*. 2024). Tolerance to high temperatures, which allows seeds to retain germination ability across a broader temperature range, is not time‐sensitive. Instead, this tolerance ensures resistance to high temperatures or facilitates establishment in areas with higher temperatures (AsheJepson *et al*. 2023), where these high‐temperature thresholds are less variable from year to year. Therefore, *T*_*c*_ is generally more stable and serves as a defence mechanism against increasing temperatures. The lower variation and noise in *T*_*c*_ response allowed clearer trend identification and more accurate predictions (Chen *et al*. 2024). In contrast, parameters like *T*_*b*_ and *GR*_*50*_ are more variable, and these fluctuations complicate their prediction (Filipe *et al*. 2023).

### Seed colour and germination cardinal temperature across geographic regions

Seeds often have lower *T*_*b*_ values in higher latitude regions, which is likely an adaptation for germination in cooler temperatures. A low *T*_*b*_ enables seeds to begin germinating as temperatures increase slightly in spring, which is advantageous in regions with shorter growing seasons (Dostatny *et al*. 2015). This adaptation supports maximum growth within the limited growing period before winter (Hoyle *et al*. 2015). These trends also varied by seed colour. In lower longitude (western) regions, seeds experience a more moderate climate with fewer temperature extremes and, therefore, may not require such high *T*_*c*_ values (Wu *et al*. 2019). Conversely, seeds from higher longitudes (eastern areas) often face more variable, continental climates and might have evolved higher *T*_*c*_ values to withstand increased temperature fluctuations and prolonged heat during summer. This adaptation allows seeds to germinate under warmer conditions, which could be beneficial in regions with hot summers (Dwyer & Erickson 2016).

The eastward increase in TGR and the spatially coherent clustering of traits align with climate‐driven selection, as the more continental southeastern regions experience more thermal variability and higher summer temperatures, conditions that favour broader germination windows (Blanco *et al*. 2014; Renzi *et al*. 2022). Cluster‐specific profiles – e.g., higher ceiling temperatures and *GR*_*50*_ in central Balkan populations versus narrower TGRs in southern Balkan groups – reflect divergent strategies, ranging from thermally flexible to more conservative thresholds, consistent with intraspecific variability arising from local adaptation (Beckie *et al*. 2012; Darmency & Fleury 2020). These patterns parallel predictive models showing shifts in dormancy and emergence under changing temperature and precipitation regimes (Chantre *et al*. 2012; Bastida *et al*. 2022). Although the positive TGR–longitude correlation does not demonstrate causation, it is consistent with trait clines along environmental gradients (Alshallash 2018). With climate change expected to alter European temperature regimes, populations possessing broader TGRs and higher thermal ceilings may have adaptive advantages (Blanco *et al*. 2014; López‐Palacios *et al*. 2022). Genotypic data and reciprocal transplant experiments will be essential to disentangle phenotypic plasticity from local adaptation.

In summary, climate conditions exert significant influence on seed traits, particularly germination temperature thresholds. These adaptive responses optimize germination timing to coincide with favourable environmental windows, thereby enhancing survival and reproductive success. Seeds originating from cooler or higher‐latitude regions typically have lower base temperatures (*T*_*b*_), enabling earlier germination to exploit shorter growing seasons. In contrast, seeds from warmer climates, or those with higher ceiling temperatures (*T*_*c*_), often delay germination, which is an apparent strategy to avoid premature exposure to heat stress or drought during early development.

## CONCLUSION

Our study provides strong evidence that germination cardinal temperatures and germination rate (*GR*_*50*_) in *A. fatua* are shaped by a complex interplay of seed morphological traits and environmental factors. Key traits, including seed mass, awn characteristics, and hairiness, differentially influenced *GR*_*50*_ and thermal thresholds (*T*_*b*_ and *T*_*c*_), while seed colour and geographic origin emerged as strong predictors of temperature responses. These trait–environment relationships likely reflect long‐term adaptive evolution, underscoring how morphological differentiation could enhance ecological fitness across diverse climate conditions and landscapes.

## AUTHOR CONTRIBUTIONS

Experiments were conducted by SV and DB, with seed collection by SV, DS, and DB. The experimental setup and measurements were performed by DS and AA. Conceptualization and study design were led by MO and SV, while data processing was carried out by MO, RP and PP. The first draft of the manuscript was written by MO, PP and RP, with all co‐authors contributing to revisions and interpretations. All authors have thoroughly reviewed the final manuscript, unanimously agreed on its content, and accept responsibility for the accuracy and integrity of the work, adhering strictly to the ICMJE criteria.

## CONFLICT OF INTEREST

The authors declare no competing interests.
